# An expanded diversity of oomycetes in Carboniferous forests: Reinterpretation of *Oochytrium lepidodendri* (Renault 1894) from the Esnost chert, Massif Central, France

**DOI:** 10.1371/journal.pone.0247849

**Published:** 2021-03-02

**Authors:** Christine Strullu-Derrien, Marc Gèze, Alan R. T. Spencer, Dario De Franceschi, Paul Kenrick, Marc-André Selosse, Andrew H. Knoll

**Affiliations:** 1 Institut Systámatique Evolution Biodiversitá, Musáum national d’Histoire naturelle, CNRS, Sorbonne Universitá, Paris, France; 2 Department of Earth Sciences, The Natural History Museum, London, United Kingdom; 3 Centre de microscopie et d’imagerie numérique du muséum, Muséum National d’Histoire Naturelle, Paris, France; 4 Département AVIV, UMR 7245 MCAM Molécules de communication et Adaptation des Micro-organismes, Musáum national d’Histoire naturelle, CNRS, Sorbonne Universitá, Paris, France; 5 Department of Earth Science & Engineering, Imperial College London, London, United Kingdom; 6 Centre de Recherche en Paléontologie Paris, Musáum national d’Histoire naturelle, CNRS, Sorbonne Université, Paris, France; 7 Department of Plant Taxonomy and Nature Conservation, University of Gdańsk, Gdańsk, Poland; 8 Department of Organismic and Evolutionary Biology, Harvard University, Cambridge, Massachusetts, United States of America; Indiana University Bloomington, UNITED STATES

## Abstract

335–330 million-year-old cherts from the Massif Central, France, contain exceptionally well-preserved remains of an early forest ecosystem, including plants, fungi and other microorganisms. Here we reinvestigate the original material prepared by Renault and Roche from collections of the Muséum National d’Histoire Naturelle, Paris, and present a re-evaluation of *Oochytrium lepidodendri* (Renault 1894), originally described as a zoosporic fungus. Confocal laser scanning microscopy (CLSM) was used to study the microfossils, enabling us in software to digitally reconstruct them in three-dimensional detail. We reinterpret *O*. *lepidodendri* as a pseudofungus and favour placement within the oomycetes, a diverse clade of saprotrophs and both animal and plant parasites. Phylogenetically, *O*. *lepidodendri* appears to belong to a group of oomycetes distinct from those previously described from Paleozoic rocks and most likely related to the Peronosporales *s*.*l*. This study adds to our knowledge of Paleozoic eukaryotic diversity and reinforces the view that oomycetes were early and diverse constituents of terrestrial biotas, playing similar ecological roles to those they perform in modern ecosystems.

## Introduction

Cherts exposed in outcrops near Esnost, France, about 15 km north of Autun, along the northern margin of the Massif Central, have long been known to preserve an exceptional record of Carboniferous vegetation (e.g. [1, 2]). Peats and other low latitude wetland sediments deposited during the late Visean Age of the lower Carboniferous Period (ca 335–330 million years ago [3]) were silicified during early diagenesis [4] exquisitely preserving biological remains in three-dimensional anatomical detail (e.g. [5]).

Renault ([1, 6–8]) described from Esnost a population of microorganisms within the xylem cells of an arborescent lycopsid, naming the new species *Oochytrium lepidodendri* and interpreting it as a chytrid fungus, based on overall morphology, dehiscence and the position of its sporangia. He considered that *O*. *lepidodendri* resembles but does not precisely match modern chytrid genera as understood in the 19th century, in particular, pointing to genera such as *Olpidium*, *Olpidiopsis*, *Rozella*, *Woronina*, and *Cladochytrium*. Not long after Renault’s original description, Seward [9] discussed *O*. *lepidodendri* in a textbook. In support of Renault’s interpretation, Seward drew specific comparison between *O*. *lepidodendri* and *Hyphochytrium*, also attributed to chytrid fungi at that time.

Molecular phylogeny has changed our view of taxa thought by Renault and Seward to resemble *O*. *lepidodendri*. *Olpidium* lies among the zoosporic true fungi (Eumycota) [10]. *Hyphochytrium* is now known to belong to the Stramenopila [11], a clade containing several groups of algae and heterotrophic protists, rather than to the true fungi [12]. More specifically, *Hyphochytridium* and closely related taxa are united with their sister group, the Oomycota [13], into the Pseudofungi, a widespread and diverse group of microorganisms that have evolved in many respects to mimic fungi in their ability to interact with plants [14].

Beginning with Renault’s (e.g. [1, 2, 6–8, 15, 16]) early studies, both Fungi [17–19] and Oomycota [20–22] have been found in the Esnost chert. Fungi [23] and Oomycota [24] have also been reported from the Combres chert, a nearby locality of the same age.

Consistent with the ancient-modern comparisons proposed a century ago, *O*. *lepidodendri* could represent any of three distinct groups within the Fungi or the Stramenopila. With this in mind, we take a fresh look at Renault’s fossils and additional materials prepared by his collaborator Auguste Roche in the hope of learning more about osmotrophic eukaryotes in ancient terrestrial ecosystems. We use a novel approach [25, 26] that combines confocal laser scanning microscopy (CLSM) with additional software methods to digitally study microfossils in 3D. Based on our reinvestigation of this material from the collections of the Muséum National d’Histoire Naturelle, Paris, we present new 3D documentation of Renault and Roche’s original specimens, provide an emended diagnosis and designate a lectotype and paralectotypes for *O*. *lepidodendri* [6], and reinterpret these fossils as oomycetes.

## Materials and methods

We examined historical collections of thin sections made by Renault and Roche toward end of the 19^th^ century, focusing on three sections that contain Renault’s original *Oochytrium* population. These are housed at the Muséum National d’Histoire Naturelle, Paris and are accessioned under the following numbers: MNHN.F.48152.0 n°43 Roche Collection; MNHN.F.45876.0 n°1145 Renault collection and MNHN.F.45877.0 n°1146 Renault collection. Transverse sections of the encompassing plant stem were photographed using a Hirox RH-2000 digital microscope. A Nikon Eclipse 80i compound microscope equipped with a Nikon D300 camera was used to examine and photograph the microorganisms under transmitted light. Focus stacking was performed on the image series using Helicon Focus 7.5.1 software (https://www.heliconsoft.com/heliconsoft-products/helicon-focus/). We employed our recent new method developed for studying fossils in thin sections, using confocal scanning laser microscopy and image processing software that allows us to produce high-resolution three-dimensional visualizations of microfossil structures, as well as video animations [25, 26]. We acquired confocal images with a Zeiss LSM 880 laser-scanning confocal microscope using a 40 X/1.3 oil DIC UV-IR objective. The Quantum efficiency (QE) of the detector was about 50%. An auto-fluorescence signal was collected with an Airyscan head using a 32 GASP detector array in super resolution mode, which increases the resolution and the SNR by a factor 1.7. Images were recorded with pixel dimensions of 90 nm. Autofluorescence of the samples was excited with the 488 nm line of argon laser (power 1%). Emission was collected with a high pass filter > 516 nm. Samples were visualised with 16-bit depth and 0.2 airy unit for each elementary detector of the Airyscan head. Typically producing 120 slice z-stacks comprising of individual focal planes, each separated by a 250 nm z step, corresponding to a z depth of 30 μm. The fluorescence signal from each z-plane was projected onto a maximum projection image. In addition, 3D volume rendered models of the specimen were generated with the 3D reconstruction and display module of the software Zen Black version 2.3 (Zeiss corporation). Furthermore, the z-stacks were imported into the volume rendering software Dragonfly v4.1.0 [27] to explore the morphology and internal contents of the microfossils. This enabled high-resolution three-dimensional digital reconstructions to be produced.Video annimations (S1 and S2 Movies) showing these reconstructions were generated in Blender (v2.83; https://www.blender.org). Digital data used for reconstructions has been deposited in a Zenodo repository (S1 Data).

## Results

### Systematics

Kingdom: Stramenopila [28], emend. [10].

Phylum: Oomycota [29].

*Oochytrium* Renault 1894 (*descriptio generico-specifica)*, emend.

*Oochytrium* B. Renault [6].

*Type species—Oochytrium lepidodendri* Renault [6]. emend. By monotypy.

*Oochytrium lepidodendri* Renault [6]. emend.

*Emended diagnosis*: structures interpreted as oogonia ovoid (ca 8–11 μm x 14–16 μm) to spheroidal (ca 13 μm in diameter), commonly containing a single oospore. Oogonia solitary or linked in a linear array by short connecting hyphae. Up to three hyphal extensions projecting from oogonium, some of which developing swellings interpreted as antheridia; cross walls visible between oogonium and antheridia. Coenocytic hyphae from a few microns to ca 15 μm long, ca 1.7μm to up to 2.5 μm in diameter when the antheridium develops. Dispersed oospores not attached to hyphae.

*Lectotype*: Since Renault [1, 6–8] did not designate a holotype, we have selected the assemblage in Fig 16 (xylem cell, middle top) from Renault [6] as the lectotype.

*Syntypes*: slides MNHN.F.48152.0 n°43, MNHN.F.45876.0 n°1145 and MNHN.F.45877.0 n°1146 from the collections at the Muséum National d’Histoire Naturelle, Paris.

*Locality*: Esnost, Massif Central, France.

*Age*: ca 330Ma, late Visean [3].

Mycobank: n° 181424 [30–32].

Description:

> Description from Renault ([7] repeated in [1, 8])–Translation from French

Renault’s description of *Oochytrium lepidodendri* [1, 7–8] within xylem cells of *Lepidodendron estonense* is as follows: The mycelium has the form of slender single or branched threads at different stages of development, the ovoid sporangia, 12–15 μm x 9–10 μm in size, occur as terminal swellings of the mycelial main thread. They can show a rostrum. Some detached sporangia of spherical shape (13 μm in diameter) are free within the cavity of the xylem cell. The ovoid sporangia are of different sizes; many are smaller, and have not reached their final size. These have been detached from the mycelial thread and are found in large numbers in some of the tracheid cells, fully filling their cavity. Some mycelia appear to have borne several sporangia, each of them being placed at the end of a thread. The mycelial threads, when preserved, consist of cells, 6 to 7 μm long, with visible septa; the cells close to the sporangium are shorter and darker and sometimes inflated. The sporangium cell wall is cuticularized, brown, with a very regular shape. One of the ends, the base, is attached to a thread of variable length; the opposite end shows an opening surrounded by a rim that seems covered by an operculum. Frequently, sporangia are fixed to the xylem cells by a short part of their mycelium. Most of the sporangia are full, sometimes the protoplasma does not leave any void within the cavity, sometimes it is contracted into a spherical mass that does not touch entirely the cell wall; granulations are visible within the protoplasma, possibly simulating a cellular network. Several sporangia are open at one end and seem to have released a trail of spores (through the rostrum). Dispersed among the sporangia, some spherical structures occur; they are of the same size but with a rougher surface.

> New description

**Fig 1:**
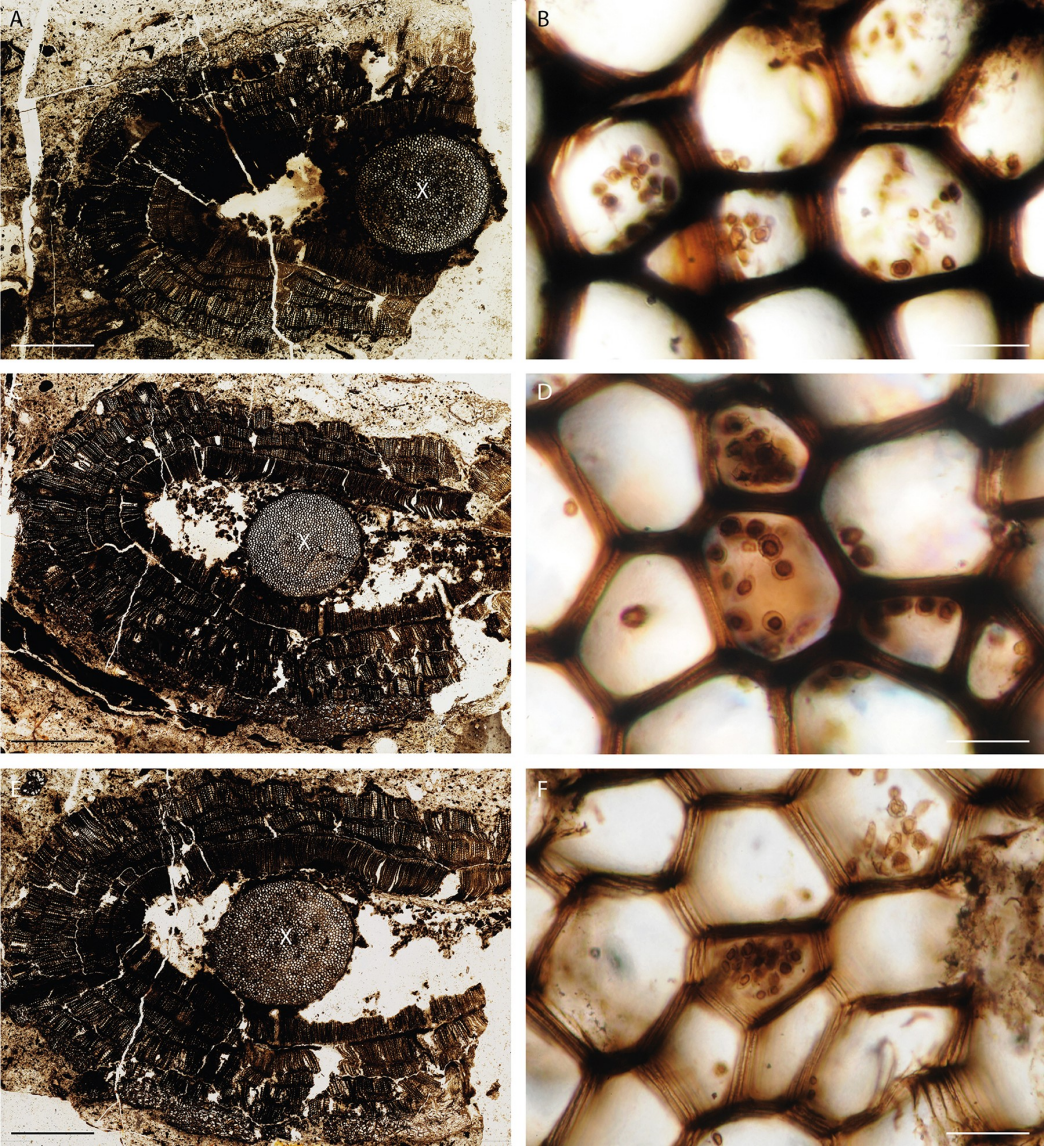
*Oochytrium lepidodendri* found within the primary xylem of *Lepidodendron estonense*. The tree branch shows thick layers of radially disposed secondary cortical tissue (A, C, E) around an inner region of the cortex poorly preserved. The primary xylem (X) is the only part of the stele represented; it contains reproductive structures of *Oochytrium esnostense* (B, D, E). In some areas the xylem cell walls are more degraded (F), this matches with the degradation stage of the inner cortex (E). Scale bars represent 2 mm in (A), 2.3 mm in (C), 1.9 mm in (E), 32 μm in (B, D), (40 μm) in (F). (A, B) MNHN.F.48152.0 n°43 Roche Collection; (C, D) MNHN.F.45876.0 n°1145 Renault collection; (E, F) MNHN.F.45877.0 n°1146 Renault collection. (A, C, E photos Gaëlle Doitteau).

**Fig 2:**
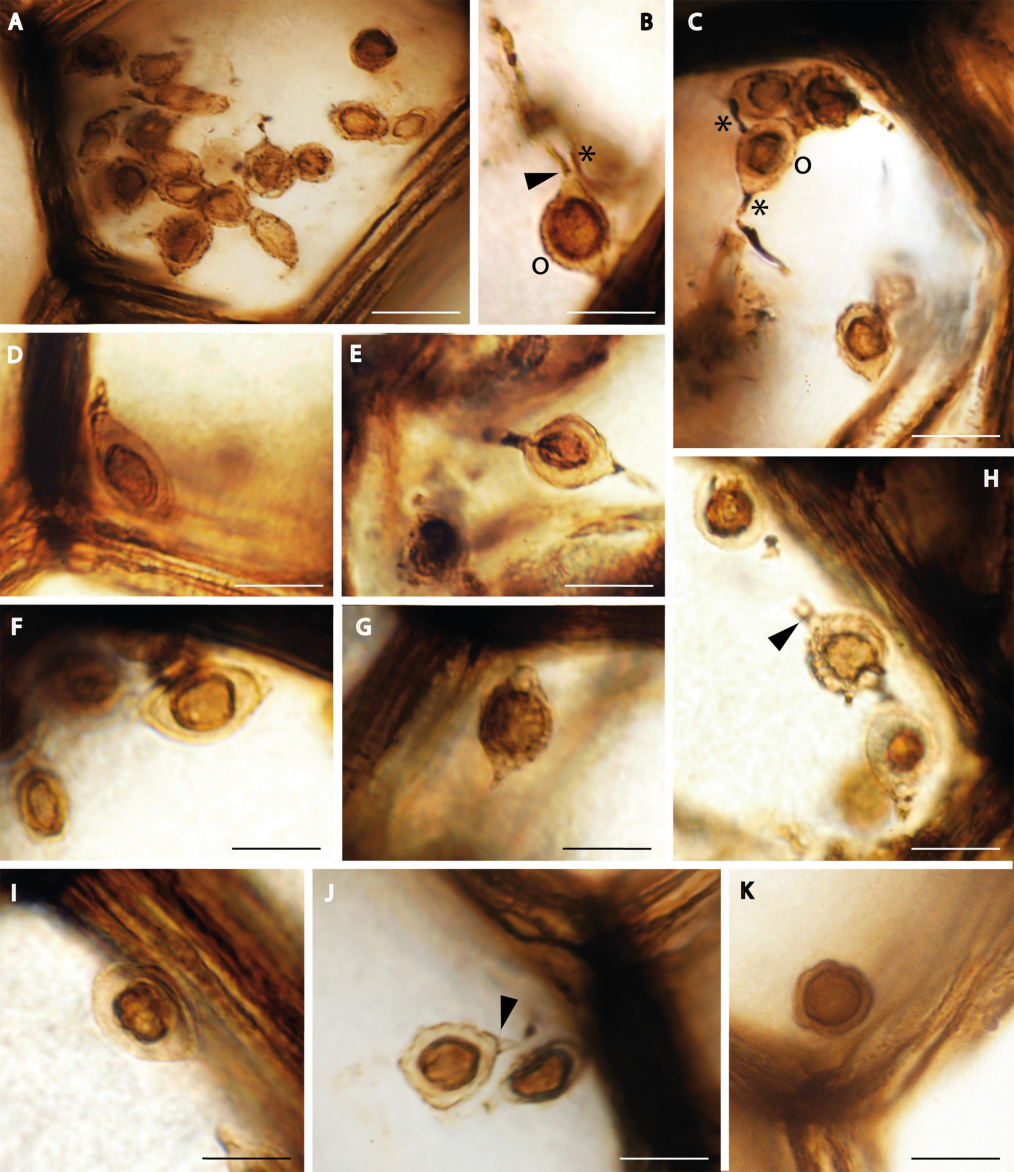
*Oochytrium lepidodendri* reproductive structures observed in light microscopy. Oogonia spheroidal to ellipsoidal discharged within the xylem cells (A). Oogonia detached because of broken hyphae (B, D, E, F, G, I, J) or arranged in short linear arrays (catenulate) (H). Cross walls visible between oogonium and antheridia (arrows in B, H, J). Hyphae developing conspicuous swellings up to 2.5 μm wide attached to the oogonia (B, C, H, J). Dispersed oospore (Fig 2K). “o” for oogonium and asterisk for antheridia. Scale bars represent 8 μm in (F, J), 9 μm in (B, I), 10 μm in (D, G, K), 11 μm in (E, H), 13 μm in (C), 18 μm in (A). (A, B, C, E, J) MNHN.F.45876.0 n°1146 Renault collection; (D, F, H, I, K) MNHN.F.45876.0 n°1145 Renault collection; (G) MNHN.F.48152.0 n°43 Roche Collection.

**Fig 3:**
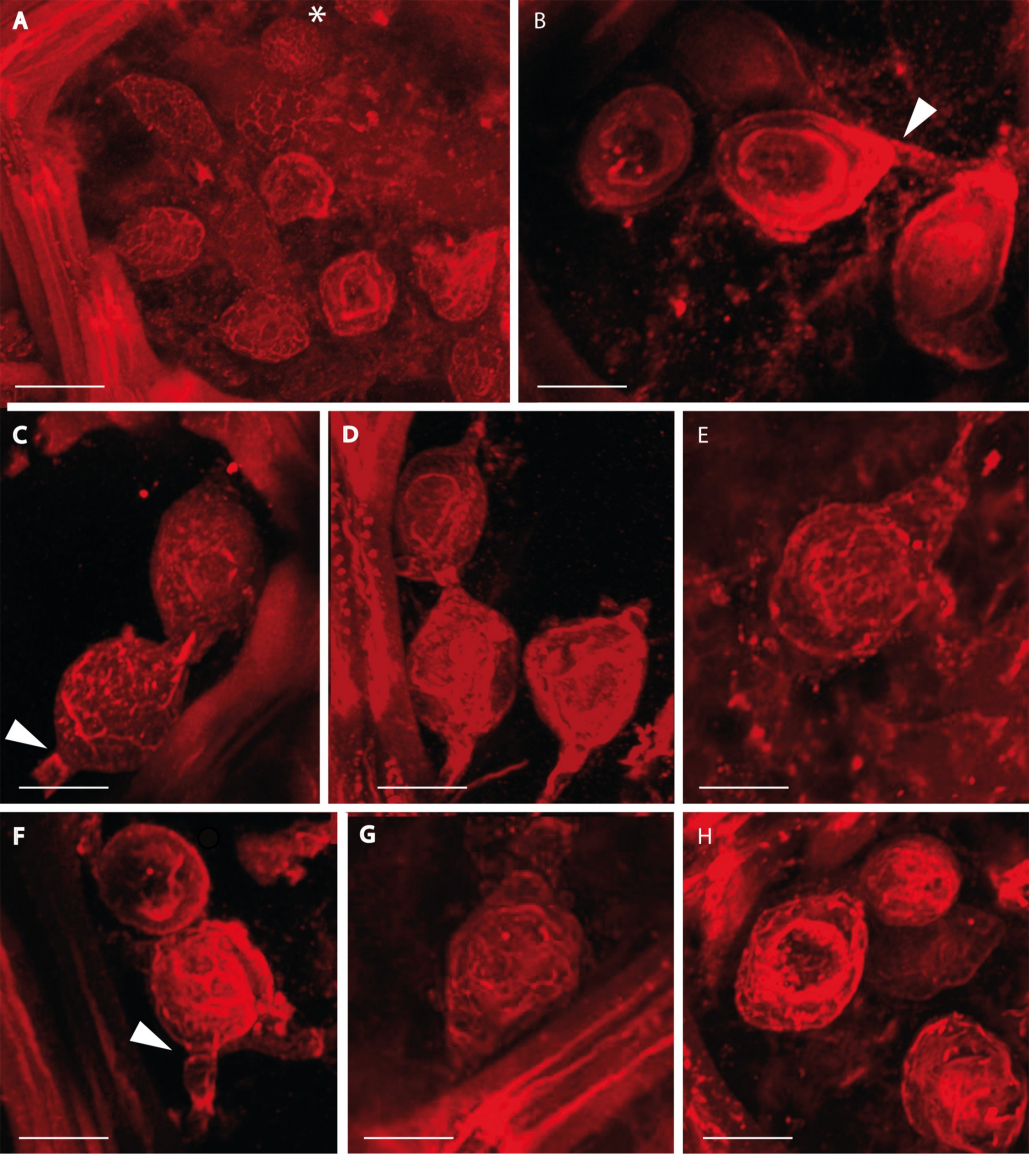
*Oochytrium lepidodendri* reproductive structures observed using confocal scanning laser microscopy. Oogonia spheroidal (asterisk in A) to ellipsoidal discharged within the xylem cells. Oogonia detached because of broken hyphae (B, E, G) or arranged in short linear arrays (catenulate) (C, D, F, H). Hyphae developing antheridia attached to the oogonium (B, C, D, F). Cross walls visible between oogonium and antheridia (arrows in B, C, F). Scale bars represent 9.5 μm in (A), 7.5 μm in (C), 7 μm in (B, H), 6 μm in (D, F, G), 5.5 μm in (E). (A, D, E, F) MNHN.F.45876.0 n°1146 Renault collection; (B, C, G, H) MNHN.F.45876.0 n°1145 Renault collection.

**Fig 4:**
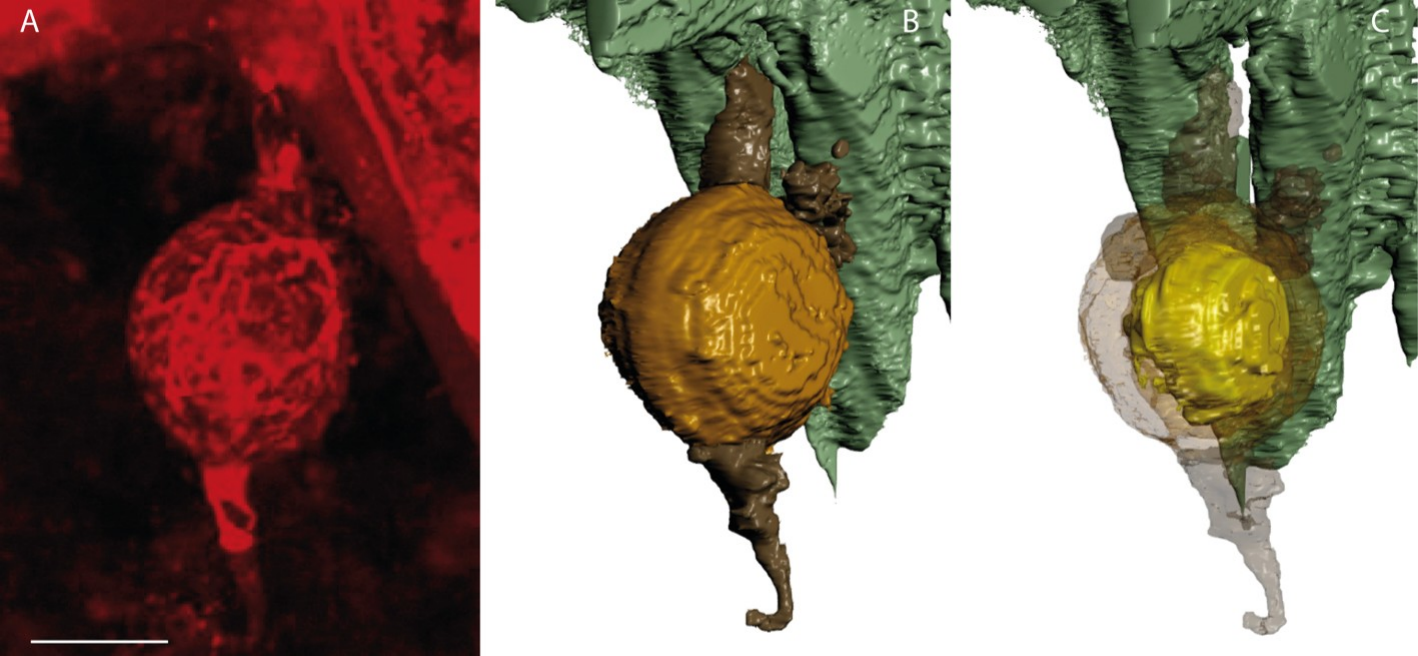
Three-dimensional reconstruction of an oogonium-antheridia complex of *Oochytrium lepidodendri* using confocal scanning laser microscopy (A) and CSLM images processed (B, C): the oospore becomes apparent by making the oogonial wall transparent (C) in the reconstruction (B). Two antheridia are attached to the oogonium possibly more (S1 Movie). No ornamentation is visible on the surface of the oogonium (B). Oogonium in light brown, antheridia in dark brown (B), oosphere in yellow (C) Scale bar represents 6.5 μm in (A). MNHN.F.45876.0 n°1146 Renault collection.

**Fig 5:**
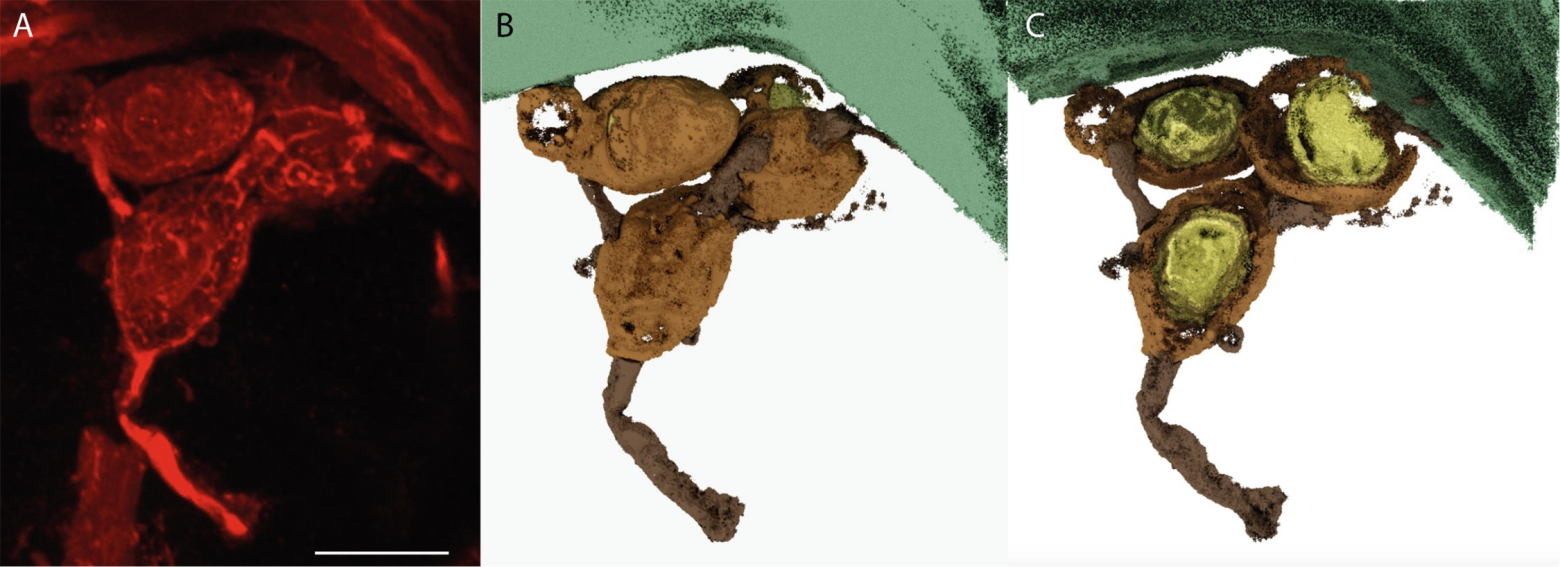
Three-dimensional reconstruction of oogonium-antheridia complexes of *Oochytrium lepidodendri* using confocal scanning laser microscopy (A) and CSLM images processed (B, C). Three antheridia are attached to one of the oogonia and possibly two others in a degraded stage (S2 Movie). Single oospore visible in the oogonium (C). Oogonium in light brown, antheridia in dark brown (B, C), oosphere in yellow (C). Scale bar represents 8.5 μm in (A). MNHN.F.45876.0 n°1146 Renault collection.

The population within the cavities of *Lepidodendron estonense* xylem cells (Fig 1) contains spheroidal (ca 13 μm in diameter) to ellipsoidal structures, ca 8–11 μm x 14–16 μm in size (Figs 2 and 3), showing zero (Figs 2A and 3A) to three filamentous extensions (Figs 2C and 5) that range from a few microns to ca 15 μm long and 1.7 to 2.5 μm wide. No branching of extensions was observed. Structures occur isolated because of broken extensions (Figs 2B, 2D, 2E, 2G, 2I, 2J, 3B, 3D, 3E and 3G) or arranged in short linear arrays (catenulate) (Figs 2H, 3C, 3D and 3H). Cross walls visible between ovoid/spheroidal structures and the extensions (Figs 2B, 2H, 2J, 3B, 3C and 3F). A distinct internal content is visible inside the ovoid structures (Figs 2–5) and this content is more or less close to the outer wall of the structure. Some extensions develop conspicuous swellings up to 2.5 μm wide attached to the spherical/ovoid structures (Figs 2B, 2C, 2H, 2J, 3B, 3C, 3E, 3F, 4 and 5). Some thick-walled structures occur dispersed within the tracheid cells (Fig 2K).

## Discussion

### Systematic affinities of *Oochytrium lepidodendri*

It was reasonable for Renault to call attention to similarities between *O*. *lepidodendri* and extant *Olpidium*. *Olpidium* commonly has elongate multinucleate (but single-celled) thalli that can differentiate into sporangia with one or more cysts. Spheroidal to ellipsoidal sporangia (with or without ornamentation) develop a single germination or discharge tube and commonly live as parasites within vascular plants, so there is both habit and habitat resonance. That noted, O. *lepidodendri* exhibits several characters that in our view eliminate *Olpidium* from further comparison: *Olpidium* species have a single discharge tube that penetrates the wall of its host cell but do not develop the multiple hypha-like extensions observed in the fossils (Figs 2C and 5). Moreover, in *Olpidium*, there are no septa bounding the spores, and, like chytrids, *Olpidium* can have multiply branching rhizoids not observed in *O*. *lepidodendri*. A small number of zoosporic fungi make structures in which hypha-like tubes connect spheroidal sporangia, but, again, these connections do not have septa that bound the sporangia.

Closer comparisons can be made between *O*. *lepidodendri* and pseudofungi. As introduced earlier, Seward [9] proposed *Hyphochytrium* as a modern analog to *O*. *lepidodendri*, believing it to be a true fungus. More recently, Krings et al. [23] reported narrow hyphae (depicted in their Fig 1I) and spore-like structures (their Fig 1M–1O) within the tissues of *Lepidodendron rhodumnense* in a chert from Combres and proposed that they might belong to *Oochytrium lepidodendri*, which they interpreted as an oomycete. Although these fossils are interesting in their own right, we believe that they are distinct from the population observed by Renault and re-examined here.

Hyphochytrids and oomcyetes are sister groups, together forming a clade called the pseudofungi that comprises osmotrophic saprotrophs and parasites found widely in marine and terrestrial environments. They differ in key characters that are not amenable to preservation: molecular sequence structure, zoospore flagellation, and cell wall chemistry [33]. Differentiating these clades based on morphology can be more challenging, especially for early branching oomycete lineages that had holocarpic thalli (i.e. the whole thallus turns into a sporangium at maturity) much like those of hyphochytrids.

A number of features support the interpretation of *O*. *lepidodendri* as belonging to the pseudofungi. In *Hyphochytrium*, up to three short hypha-like structures can extend from sporangia (e.g. [34]), much as observed in the Esnost fossils. However, the structures observed in our material show a distinct thick-walled content that does not develop in *Hyphochytrium* sporangia (see Fig 1 from [13]). We therefore reinterpret Renault’s ovoid sporangia as oogonia. Connections between oogonia in catenulate thalli (Fig 3D) approximate the septate hyphal connections observed in modern species. The oogonia contained thick-walled oospores (Figs 4 and 5). The oospore becomes apparent by making the oogonial wall transparent (Fig 4C) in the reconstruction of an oogonium preserved in 3D (Fig 4A and 4B and S1 Movie). No ornamentation is visible on the surface of the oogonium (Figs 4B and 5C). Antheridia are more difficult to illustrate because of the small size of the hyphae compared, for example, to those observed in other Carboniferous oomycetes (e.g. [23, 35]). Also, they are ephemeral, not always persisting to maturity in most species. However, some extensions develop conspicuous swellings up to 2.5 μm wide attached to the spherical/ovoid structures (Figs 3B, 3C, 3E, 3F, 4 and 5 and S1 and S2 Movies) and a cross wall has been observed in several of them (Figs 2B, 2H, 3C, 3F, 4 and 5); it resembles the cross wall that appears in connection with the formation of reproductive organs in some extant oomycetes (such as *Pythium* spp.). Acknowledging these difficulties, we interpret these swollen structures as antheridia.

Oogamous sexual reproduction is a morphological character which serves to identify oomycetes [36]; this is the formation of an oogonium in which there is cleavage or rearrangement of the oogonial protoplasm to form one or more oospheres. One or more antheridia develop in contact with the oogonium and this is followed by the development of a fertilization tube from the antheridium which penetrates the oogonium. Similar structures–ascogonia and antheridia–are formed by ascomycete and basidiomycete fungi but they are morphologically distinct and we did not find in our material any characters that might attach the microorganism to Dikarya. Thus, the occurrence of oogonium/antheridium complexes leads us to place *O*. *lepidodendri* within the oomycetes.

### Insights from molecular clocks and other fossils

Over the past decade, several molecular clock studies have included oomycetes. Brown and Sonrhannus [37] used oomycetes as an outgroup to the more extensively sampled photosynthetic stramenopiles (ochrophytes), estimating that the two clades diverged during the late Neoproterozoic Era, with large uncertainly. The three oomycetes sampled all fall within a single order, so the analysis does not illuminate divergences within the pseudofungi. As part of a large-scale analysis of eukaryotic diversity, Parfrey *et al*. [38] included two oomycete species. Like Brown and Sorhannus [37], they estimated that oomycetes and photosynthetic stramenopiles diverged during the Neoproterozoic Era. Because the sampled oomycetes fall into two major clades, the saprolegnians and peronosporaleans, Parfrey *et al*. [38] clocks suggest a Carboniferous divergence between the major clades of derived oomycetes, consistent with a radiation of terrestrial oomycetes as forests expanded over the land surface, but again with large uncertainty. In contrast, Matari and Blair [39] estimated the divergence between oomycetes and ochrophytes as mid-Paleozoic, ca 400 million year ago as the Devonian Period began, and the saprolegnian–peronosporalean split at ca 200 million years. These analyses lack internal calibration points, do not including early diverging oomycetes, and have large uncertainties, but they emphasize the need to consider possible extinct stem groups as well as crown group oomycete clades.

Turning to the fossil record, the oldest terrestrial oomycete that has been identified based on oogonium–antheridium complexes is *Hassiella monospora* from the 407-million-year-old Rhynie chert [40]. Other oomycete-like oogonia [41, 42] are also present at Rhynie. Oomycete fossils have also been identified within the tissues of three groups of Carboniferous plants: lycophytes, ferns and pteridosperms [21, 35], occurring either in cortical tissue [23, 24, 35] or within sporangia [43]. Additional oomycete fossils occur within degraded plant fragments [20]. While all of these fossils are accepted as oomycetes, none have been placed within a specific order. Thus, fossils show that crown group oomycetes existed at the time of Esnost sedimentation, although they leave open multiple finer-scale phylogenetic interpretations of *Oochytrium lepidodendri*. We note that all Paleozoic oomycetes described to date have oogonia larger than those of *O*. *lepidodendri*. Moreover, these oogonia have an ornamented surface, sometimes with prominent extensions that do not directly conform to any ornamentation known in extant oomycetes.

Thus, as noted above, *Oochytrium lepidodendri* appears to belong to a group of oomycetes different from those previously described from Paleozoic rocks. Its oogonia and oogonium-antheridium complexes resemble similar structures observed, for example, in extant *Pilasporangium* (a genus segregated from *Pythium sensu lato* [44], Fig 5C) and some species of *Pythium per se*, including *P*. *emineosum* ([45], Fig 3H) and *P*. *camurandrum* ([45], Fig 5F). While acknowledging uncertainties, the presence of antheridia and the similarity of both oogonia and antheridia to those of some extant peronosporaleans *sensu lato* lead us to favour placement of *O*. *lepidodendri* among stem or crown group in peronosporaleans *sensu* Beakes *et al*. [46].

### Ecology

Rex [4] reported that the permineralized plants in Esnost were preserved within several distinct facies, each having a specific floral association and a distinct pattern of mineralization. *Lepidodendron esnostense* was found as large fragments in “Facies 8: *Diplobasis* chert”, a hard, massive chert, usually yellow or red in colour, in complete contrast with the lithology of other facies. Leaves, megaspores and poorly preserved fragments of stigmarian rootlets have been found in association with these large stems. *Lepidodendron esnostense* has never been encountered in growth position. Renault [1] considered that the original stem would have had a diameter of 18 cm, meaning that *L*. *esnostense* must have been arborescent.

Molecular phylogenies indicate that the oomycetes first evolved in marine environments, gaining the land in concert with the emergence of land plants and the ecosystems they support [35, 46]. Extant marine oomycetes are parasitic; the diverse, and derived, clade of terrestrial oomycetes is divided into a largely saprophytic and parasitic saprolegnian subclade and a mostly parasitic peronosporalean group [46]. Thus, within the oomycetes, saprophytism is derived, although it has proved an effective strategy in terrestrial ecosystems.

Most known Devonian and Carboniferous oomycetes apparently lived as saprotrophs; indeed, only one Carboniferous form has been identified as parasitic [35]. *Oochytrium lepidodendri* is the first oomycete to be documented in xylem as opposed to other tissues (Table 1), occurring within the primary xylem of a water-logged branch of *L*. *esnostense* as it began to decay within the swamp (Fig 1). This observation might suggest that *O*. *lepidodendri* was saprotrophic; however, phylogenetic comparison to extant peronosporalean oomycetes tilts toward parasitism. In fact, as xylem cells are non-living by the time they become functional, the occurrence *O*. *lepidodendri* within the empty lumens of tracheids makes interpretation of feeding ecology difficult. Most of the extensions on fossilized individuals appear as broken hyphae and we have observed no attachment of the structures to xylem cell walls, suggesting the microorganisms were likely discharged into the empty cells of decaying xylem.

**Table 1:** Comparison between oomycetes described from Devonian and Carboniferous times.

Fossil	Age	Occurrence Of oogonia	Oogonium-antheridium complexes	Size & Ornamentation of the Oogonium	Ecological status	Reference
*Hassiella monospora*	407 Ma	+	+	Up to 28 μm in diameter ; ornamented by verrucae that form a reticulate pattern	Saprotroph, within degraded plant material	[40]
*Frankbaronia polyspora*	407 Ma	+	-	< or = 50 μm wide or in diameter ; smooth or with irregularly distributed conical or column-like hollow projections	Saprotroph, in plant litter and microbial mats	[41]
*Frankbaronia velata*	407 Ma	+	-	Up to 100 μm in diameter ; enveloped in prominent sheath; periphery of sheath smooth to irregularly wrinkled	Saprotroph, within a microbial mat dominated by filamentous bacteria or cyanobacteria	[42]
Unnamed	330 Ma	+	+	Up to 34 μm in diameter ; prominent ornament of long thread-like extensions that can be once or several times furcate	Saprotroph, within an accumulatio of degraded land plant and other fungal fragments	[20]
*Combresomyces cornifer*	330 Ma	+	+	< 40 μm in diameter; hollow papillations with multi-branched, antler-like extensions	Saprotroph in the periderm of the plant *Lepidodendron*	[24]
*Combresomyces williamsonii*	315 Ma	+	+	90 to 130 μm in diameter; conspicuous projections with two extensions, which sometimes dichotomize once at the tips	Parasite in the stem cortex of the plant *Lyginopteris*	[35]
*Galtierella biscalithecae*	305 Ma	+	+	Up to 20 μm in diameter; ornament of short bristles or hairs	Saprotroph in partially degraded sporangia	[43]
*Annelaurea excomis*	330 Ma	+	-	< 70 μm in diameter; hollow papillations that vary in size and shape	Saprotroph, within partially degraded plant fragments and spores	[22]
*Oochytrium lepidodendri*	330 Ma	+	+	Ca 13 μm in diameter or up to 16 μm wide unornamented	Saprotroph or parasite within tracheid cells of the plant *Lepidodendron*	[1, 6–8]; this paper

## Conclusions

*Oochytrium lepidodendri* adds to a growing list of oomycetes that lived within plant tissues in early terrestrial ecosystems. Morphological comparisons with *Pythium* and closely related taxa suggest that *O*. *lepidodendri* likely falls within the Peronosporales *s*.*l*.; its ecological role is unclear although its phylogenetic attribution suggests parasitism. In truth, there is uncertainty in both phylogenetic and functional extrapolation from extant taxa, which may not fully illuminate extinct and possibly stem group taxa in Paleozoic rocks. With only a single exception, Carboniferous oomycetes described to date are saprotrophs. Does that mean that saprophytism was prominent among Carboniferous oomycetes? Or only that the fossil record is biased toward saprotrophs because of the environments in which they lived? Only more fossils will answer this question.

## Supporting information

S1 Movie Three-dimensional animated rendering of specimen MNHN.F.45876.0 n°1146 Renault collection showing a spheroidal oogonium with two attached antheridia. Several other hyphae project up to the tracheid cell wall. Scale bar 10 μm. [4.7 MB;.mp4 (H.264) format; 1920x1080px]. Movie of Fig 4B.
(MP4)

S2 Movie Three-dimensional animated rendering of specimen MNHN.F.45876.0 n°1146 Renault collection showing four ovoidal oogonia. Three antheridia are attached to one of the oogonia. Single oospores are visible within the oogonium. Several hyphae project up to the tracheid cell wall. Scale bar 10 μm. [7.4 MB;.mp4 (H.264) format; 1920x1080px]. Move of Fig 5C.
(MP4)

S1 Data All digital data used in producing these three-dimensional models based on the confocal tomographic data is stored and available on Zenodo: https://doi.org/10.5281/zenodo.4522235. Included in the repository are the native confocal output files, extracted.bmp and.tiff format z-stacks, Dragonfly session data containing the segmentation information,.stl mesh files exported from Dragonfly, and videos created from the data.
(DOCX)
