# Supplementary material for: An expanded diversity of oomycetes in Carboniferous forests: Reinterpretation of Oochytrium lepidodendri (Renault 1894) from the Esnost chert, Massif Central, France
Source: PLoS One. 2021 Mar 2;16(3):e0247849. doi: 10.1371/journal.pone.0247849 (PMC7924773; doi:10.1371/journal.pone.0247849)
Supplement: S1 Data — Included in the repository are the native confocal output files, extracted.bmp and.tiff format z-stacks, Dragonfly session data containing the segmentation information,.stl mesh files exported from Dragonfly, and videos created from the data. (DOCX) [file pone.0247849.s003.docx]

S1 Data. All digital data used in producing these three-dimensional models based on the confocal tomographic data is stored and available on Zenodo: [https://doi.org/10.5281/zenodo.4522235](https://eur03.safelinks.protection.outlook.com/?url=https%3A%2F%2Fdoi.org%2F10.5281%2Fzenodo.4522235&data=04%7C01%7Cc.strullu-derrien%40nhm.ac.uk%7C389335889e0148db965808d8cd181730%7C73a29c014e78437fa0d4c8553e1960c1%7C1%7C0%7C637484850643457230%7CUnknown%7CTWFpbGZsb3d8eyJWIjoiMC4wLjAwMDAiLCJQIjoiV2luMzIiLCJBTiI6Ik1haWwiLCJXVCI6Mn0%3D%7C2000&sdata=q3MYDD2738aj8IzOzL2C2mUU3vPZcpV%2B2Om9Kgc3%2BLI%3D&reserved=0). Included in the repository are the native confocal output files, extracted .bmp and .tiff format z-stacks, Dragonfly session data containing the segmentation information, .stl mesh files exported from Dragonfly, and videos created from the data.
